# Genetic Panel Screening of Nearly 100 Mutations Reveals New Insights into the Breed Distribution of Risk Variants for Canine Hereditary Disorders

**DOI:** 10.1371/journal.pone.0161005

**Published:** 2016-08-15

**Authors:** Jonas Donner, Maria Kaukonen, Heidi Anderson, Fredrik Möller, Kaisa Kyöstilä, Satu Sankari, Marjo Hytönen, Urs Giger, Hannes Lohi

**Affiliations:** 1 Genoscoper Laboratories Oy, Helsinki, Finland; 2 Research Programs Unit—Molecular Neurology, University of Helsinki, Helsinki, Finland; 3 Department of Veterinary Biosciences, University of Helsinki, Helsinki, Finland; 4 Folkhälsan Institute of Genetics, Helsinki, Finland; 5 Department of Equine and Small Animal Medicine, University of Helsinki, Helsinki, Finland; 6 Section of Medical Genetics, University of Pennsylvania, Philadelphia, Pennsylvania, United States of America; University of Sydney Faculty of Veterinary Science, AUSTRALIA

## Abstract

**Background:**

The growing number of identified genetic disease risk variants across dog breeds challenges the current state-of-the-art of population screening, veterinary molecular diagnostics, and genetic counseling. Multiplex screening of such variants is now technologically feasible, but its practical potential as a supportive tool for canine breeding, disease diagnostics, pet care, and genetics research is still unexplored.

**Results:**

To demonstrate the utility of comprehensive genetic panel screening, we tested nearly 7000 dogs representing around 230 breeds for 93 disease-associated variants using a custom-designed genotyping microarray (the MyDogDNA® panel test). In addition to known breed disease-associated mutations, we discovered 15 risk variants in a total of 34 breeds in which their presence was previously undocumented. We followed up on seven of these genetic findings to demonstrate their clinical relevance. We report additional breeds harboring variants causing factor VII deficiency, hyperuricosuria, lens luxation, von Willebrand’s disease, multifocal retinopathy, multidrug resistance, and rod-cone dysplasia. Moreover, we provide plausible molecular explanations for chondrodysplasia in the Chinook, cerebellar ataxia in the Norrbottenspitz, and familiar nephropathy in the Welsh Springer Spaniel.

**Conclusions:**

These practical examples illustrate how genetic panel screening represents a comprehensive, efficient and powerful diagnostic and research discovery tool with a range of applications in veterinary care, disease research, and breeding. We conclude that several known disease alleles are more widespread across different breeds than previously recognized. However, careful follow up studies of any unexpected discoveries are essential to establish genotype-phenotype correlations, as is readiness to provide genetic counseling on their implications for the dog and its breed.

## Introduction

Novel mutations that underlie canine Mendelian disorders and traits are described at an accelerating pace, with 199 such variants currently described in the OMIA database [1]. Accordingly, the number of hereditary diseases and genetic predispositions that a dog could potentially be tested for is steadily increasing. For some breeds, up to ten breed-relevant DNA tests are commercially available through different laboratories [2,3], posing a burden on dog owners, breeders, and clinicians seeking information and pursuing comprehensive health screening. Therefore, there is a need for accurate and comprehensive, cost-effective and easy to use DNA screening solutions that provide breeders with results for several breed relevant disorders with one sampling, analysis and reporting effort. Furthermore, such screening holds great potential to serve as a discovery and diagnostic tool for researchers and veterinary clinicians.

Several canine genetic disorders such as progressive retinal atrophies (PRAs; [4]), pyruvate kinase deficiency [5], cystinurias [6] and spinocerebellar ataxias [7,8] are genetically heterogeneous, which means that multiple simultaneous genetic tests of variants in the same gene, or in different genes, are needed to understand their background across and within pure breeds. It is also nearly impossible to know in advance which DNA tests apply to a mixed breed dog. Genetic panel screening solutions that facilitate comprehensive molecular diagnostics and help exclude known disease-causing mutations are therefore expected to support pet health care and research. Testing for other mutations, besides the known breed-relevant ones, also has the potential to advance knowledge on breed prevalence of disease-linked variants in dogs. Initial research discovery studies are often limited to one or a few breeds, leaving the true breed and geographic distribution of a new disease-associated variant incompletely explored. Although many mutations are considered breed specific [3], others such as the ones underlying degenerative myelopathy (DM; [9]), hyperuricosuria (HUU; [10]) or factor VII (FVII) deficiency [11,12] are thought to be ancient in their origins and therefore potentially widespread across breeds. Shared breed ancestry is also reflected in the disease heritage of breed groups, with examples including primary lens luxation (PLL; [13]) in the terrier breed group, and collie eye anomaly (CEA; [14]) in collie-like herding breeds. Inadvertent or planned crossbreeding may further complicate the distribution pattern of disease-causing variants.

We report here on the experience gained by an initial DNA panel screening feasibility evaluation, testing for 93 variants underlying canine inherited disorders in nearly 7000 dogs representing more than 230 breeds. Our findings illustrate the power of such a comprehensive genetic approach as a diagnostic platform and research discovery tool providing insights into the distribution of disease alleles and inherited disorders across canine breeds. We further demonstrate the clinical presentation and relevance of several of the novel discoveries for the newly affected breeds. More broadly, our study highlights general issues related to high fidelity multiplex testing, such as the need for careful genetic and clinicopathological follow up of unexpected findings, maintenance of close ties with the research and dog community, and appropriate genetic counseling.

## Materials and Methods

### Animals and samples

All canine buccal swab or EDTA blood samples used for multiplex screening (N = 6788) were originally submitted for commercial DNA testing at Genoscoper Laboratories (Genoscoper Laboratories Oy, Helsinki, Finland) during 2013 to 2015. The majority of the study sample consisted of Finnish dogs (69.5%), while samples from Russia (5.5%), the Netherlands (4.8%), Germany (3.5%), the United States (2.7%), Great Britain (2.0%), France (1.9%) and Norway (1.0%) represented other major subgroups. There were 233 different dog breeds represented among the studied dogs, with 156 breeds accounted for with ≥5 individuals (S1 Table). The majority of the examined dogs (N = 4668; 68.8%) represented breeds with at least one prior known breed-relevant DNA test included among the tested genetic variants. The breed of a dog was reported by its owner with accompanying registration information confirming the dog as being registered under Fédération Cynologique Internationale (FCI) or American Kennel Club (AKC)- standards. This study was ethically approved by the Animal Ethics Committee of the State Provincial Office of Southern Finland, Hämeenlinna, Finland (permit number: ESAVI/6054/04.10.03/2012). All dog owners provided written consent for the use of their dog’s DNA sample for research purposes.

Follow up samples of 233 Brazilian Terriers genotyped for the canine multifocal retinopathy (*cmr1*) variant [15] only, and 41 Norrbottenspitzes genotyped for the Finnish Hound cerebellar ataxia variant [16] only, were selected from the biobank of the canine genetics research group at the University of Helsinki.

### Multiplex panel design and validation

A custom genotyping microarray for selected canine disease-associated variants (S2 Table) was developed based the robust and widely utilized Illumina Infinium HD Ultra platform (Illumina, Inc., San Diego, CA, USA), and validated as the MyDogDNA® test through Genoscoper Laboratories Oy, Helsinki, Finland [17]. In brief, a comprehensive literature and online database review [1,2] was first performed to identify and compile template sequence information on target mutations underlying canine inherited disorders. Second, genotyping assays were designed according to the manufacturer’s guidelines (Illumina, Inc.) to directly target known point mutations, insertions and deletions. Each disease marker was replicated three times in the array design. Finally, the accuracy and reproducibility of the panel test results was validated with a set of experiments [17] addressing source sample type, internal replicate concordance, test-retest reproducibility, and with evaluation by an independent DNA testing laboratory (Antagene, France) submitting a selection of samples with known disease genotypes for blind testing. Specific disease assays were validated by 1) genotyping of samples with genotypes known *a priori*; 2) sequencing validation of identified disease genotypes *a posteriori*; 3) synthetic oligonucleotides for rare conditions where no control samples were available.

### Genotyping

Microarray analyses were carried out following manufacturer-recommended standard protocols (Illumina, Inc., San Diego, CA, USA). All genotype data from samples with call rates below 95% of the analyzed markers was discarded to ensure high quality data, and all genotype calls were manually evaluated.

All mutant allele findings in unexpected breeds were further confirmed by standard capillary sequencing on an ABI3730xl DNA Analyzer instrument (Thermo Fisher Scientific, Inc., Waltham, MA, USA) at the Sequencing Unit of the Finnish Institute of Molecular Medicine (FIMM). Sequencing primers for each investigated disease variant are listed in S3 Table. PCR reactions were performed on ~20 ng of genomic DNA using Amplitaq Gold Master Mix (Applied Biosystems, Waltham, MA, USA) with reactions initiated at 95°C for 5 min, followed by 34 cycles of 30 s at 95°C, 30 s at 64°C and 45 s at 72°C, and finally 10 min at 72°C. Pre-sequencing PCR product clean-up was performed enzymatically using Exonuclease I (Exo1) and FastAP™ Thermosensitive Alkaline Phosphatase (Thermo Fisher Scientific) according to the manufacturer’s protocol. Additional separate genotyping for *cmr1* in Brazilian Terriers took place using KASP genotyping chemistry according to the manufacturer’s assay design (LGC Ltd., Middlesex, UK). Additional separate genotyping for Finnish Hound cerebellar ataxia in the Norrbottenspitz was carried out on a LightCycler 480 II instrument (Roche Diagnostics, Basel, Switzerland) using standard probe hydrolysis detection chemistry with LC480 Probe Master Mix (Roche Diagnostics), and the following primers: 5’-CGTAGACTACGAGACTGCATTTATTCA-3’ (forward) and 5’-GATTAAACATAGCTTGTGCACTGTGT-3’ (reverse); and probes: 5’-TGCTGCTCAGATGCTA-3’ (VIC) and 5’-TGCTGCTCAGGTGCTA-3’ (FAM).

### Coagulation factor VII deficiency measurements

We collected citrated blood samples for coagulation analysis from a seven-year-old Welsh Springer Spaniel female and her litter of six dogs, approximately two and a half years old at the time of this study. The carrier female and her litter represented all genotypes (N_normal_ = 1, N_carrier_ = 3, N_affected_ = 3) of a variant (*F7* c. c.407G>A) predisposing to factor VII deficiency. Routine coagulation screening tests carried out included measurement of prothrombin and partial thromboplastin times (Movet Oy, Kuopio, Finland) as well as specific coagulation factor activity analyses using factor deficient plasma as previously described [11].

### Urine uric acid measurements

We evaluated manifestation of hyperuricosuria in fourteen Lagotto Romagnolo dogs representing all genotypes (N_normal_ = 7, N_carrier_ = 3, N_affected_ = 4) of a predisposing variant (*SLC2A9* c.616G>T). The sample consisted of eight females and six males with a median age of one year and one month at the time of the study (range ten months to seven years). Hyperuricosuria was evaluated based on a previously described urine test [18], evaluating urine uric acid: creatinine concentration ratios. Fresh midstream catch urine samples were used for the determination. Uric acid was analyzed by an enzymatic method, and creatinine by a kinetic picric acid method, using the reagents and adaptations of an automatic chemistry analyzer (Konelab 30i, Thermo Fisher Scientific Oy, Vantaa, Finland).

### Ophthalmology examinations

Ophthalmology examinations in Brazilian Terriers were performed by an ECVO (European College of Veterinary Ophthalmologists) panelist or an ECVO diplomate. The examinations included ophthalmoscopy, biomicroscopy and funduscopy, using tropicamide as the mydriatic. An Optical Coherence Tomography (OCT) examination of one dog homozygous for the *cmr1* mutation (*BEST1* c.73C>T) was carried out at the HUS Eye and Ear Hospital (Helsinki, Finland) using an OCT Plus SPECTRALIS device (Heidelberg Engineering GmbH, Heidelberg, Germany). No mydriatic was used for the OCT examination.

### Statistical and pedigree analyses

The statistical significance of observed differences in phenotype values between groups of dogs was evaluated by standard unpaired t-tests and one-way analysis of variance (ANOVA). Pedigrees were drawn using GenoPro® genealogy software [19], based on information retrieved from the Finnish Kennel Club registry database [20].

## Results

### Microarray-based panel testing yields high fidelity results

We developed a custom-designed Illumina Infinium technology-based bead chip SNP genotyping microarray to screen dogs for 93 different genetic variants underlying a wide variety of canine hereditary disorders and genetic predispositions (S2 Table). The performance of the microarray was extensively validated as also described elsewhere [17]. Blood and buccal swab samples performed equally well on the array (median marker call rates of 98.1% vs 98.2%, respectively). Internal replicate and test-retest (reproducibility) concordances were 99.8%. Only markers reaching 100% sensitivity and specificity upon testing with control genotypes, or validated by an experiment with a synthetic control oligonucleotide, were considered for reporting.

### High overall carrier frequency of known mutations across breeds

For this study, we screened samples from 6788 dogs representing 233 breeds between 2013 and 2015. Mutant alleles of well-known hereditary disease traits were encountered in breeds as expected based on current literature and database information. Carrier frequencies for known breed-relevant disorders are listed in S1 Table for breeds represented by at least 30 dogs. Based on the examined set of 93 disease variants, we observed that 17.8% (N = 1208) of the tested dogs carried at least one of the tested variants, while 2.5% (N = 170) were genetically affected for a disease condition. The maximum number of disorders carried by any individual dog was three (observed in three dogs). Two disorder variants were carried by 1.0% (N = 66), and one disorder variant by 16.8% (N = 1139) of the tested dogs.

The genetic panel screening approach revealed 15 known disease-linked variants in an additional 34 breeds in which they have not previously been documented in the peer-reviewed literature (Table 1). Each mutant allele finding in an additional breed was confirmed by a second genetic technology, Sanger sequencing. After confirming the genetic presence of a mutation in an additional breed, it is crucial to establish a genotype-phenotype correlation by exploring whether the risk variant phenotypically manifests on a different genetic background. In this study, clinical validation was initiated with owner interviews regarding clinical manifestations, and where possible, continued by recruitment of genetically affected dogs for relevant clinicopathological examinations as described in the following sections.

**Table 1:** Summary of disease variant findings in additional breeds.

Disorder	Gene and mutation	Previously reported breeds	Additional breed(s) with N observed carriers
Catalase Deficiency	*CAT* c.979G>A (p.Ala327Thr)	Beagle	American Foxhound: 1/1 (100%)
Chondrodysplasia (Dwarfism)	*ITGA10* c.2083C>T (p.Arg695*)	• Norwegian Elkhound, grey • Karelian Bear Dog	Chinook: 5/47 (10.6%)
Craniomandibular Osteopathy (CMO)	*SLC37A2* c.1332C>T (p.Asp444Asp)	• Cairn Terrier • Scottish Terrier • West Highland White Terrier	• Australian Shepherd: 1/140 (0.7%) • Lancashire Heeler: 1/40 (2.5%)
Factor VII (FVII) Deficiency	*F7* c.407G>A (p.Gly136Glu)	• Alaskan Klee Kai • Airedale Terrier • Beagle • Giant Schnauzer • Scottish Deerhound	• American Foxhound: 1/1 (100%) • Finnish Hound: 54/237 (22.8%) • German Wirehaired Pointer: 1/3 (33.3%) • Irish Water Spaniel: 1/3 (33.3%) • Japanese Spitz: 1/6 (16.7%) • Miniature Schnauzer: 1/7 (14.3%) • Papillon/Phalene: 5/41 (12.2%) • Sealyham Terrier: 1/1 (100%) • Welsh Springer Spaniel: 18/76 (23.7%)
Familial Nephropathy (FN)	*COL4A4* c.115A>T (p.Lys40*)	• American Cocker Spaniel • English Cocker Spaniel	Welsh Springer Spaniel: 10/76 (13.2%)
Hyperuricosuria (HUU)	*SLC2A9* c.616G>T (p.Cys188Phe)	• Black Russian Terrier • Dalmatian • English Bulldog • >10 other breeds	• Danish-Swedish Farmdog: 13/168 (7.7%) • German Hunting Terrier: 3/22 (13.6%) • Kromfohrländer: 1/160 (0.6%) • Lagotto Romagnolo: 52/637 (8.2%) • Spaniel de Pont-Audemer: 1/4 (25%) • Swedish Vallhund: 3/71 (4.2%)
Multi-drug resistance 1 (MDR1)	*ABCB1* c.295_298delAGAT (p.Asp75*fs*)	• Australian Shepherd • Collie • Shetland Sheepdog • >10 other breeds	• Chinook: 7/47 (14.9%) • Danish-Swedish Farmdog: 2/168 (1.2%)
Multifocal Retinopathy 1 (*cmr1*)	*BEST1* c.73C>T (p.Arg25*)	• Australian Shepherd • Bulldog • Great Pyrenees • Mastiff • >10 other breeds	Brazilian Terrier: 25/107 (23.4%)
Multifocal Retinopathy 3 (*cmr3*)	*BEST1* c.1388delC (p.Pro463*fs*)	Lapponian Herder	Finnish Lapphund: 3/218 (1.4%)
Myotonia Congenita	*CLCN1* c.2665insA (p.Arg889*fs*)	Australian Cattle Dog	Border Collie: 2/29 (6.9%)
Primary Lens Luxation (PLL)	*ADAMTS17* c.1473+1G>A	• American Hairless Terrier • Chinese Crested • Jack Russell Terrier • >20 other breeds	Danish-Swedish Farmdog: 37/168 (22.0%)
Progressive Early-Onset Cerebellar Ataxia	*SEL1L* c.1972T>C (p.Ser658Pro)	Finnish Hound	Norrbottenspitz: 13/103 (12.6%)
Rod-Cone Dysplasia 3 (rcd3)	*PDE6A* c.1940delA (p.Asn616*fs*)	• Welsh Corgi Cardigan • Welsh Corgi Pembroke	• Chinese Crested: 1/48 (2.1%) • Pomeranian: 1/8 (12.5%)
von Willebrand's disease, type I	*VWF* c.7437G>A (p.Ser2479Ser)	• Bernese Mountain Dog • Doberman • Manchester Terrier • >10 other breeds	• Barbet: 2/39 (5.1%) • Brazilian Terrier: 1/107 (0.9%) • Dutch Shepherd Dog—Longhaired: 1/16 (6.3%)Kromfohrländer: 13/160 (8.1%)
von Willebrand's disease, type II	*VWF* c.4937A>G (p.Asn1646Ser)	• German Shorthaired Pointer • German Wirehaired Pointer	• Barbet: 1/39 (2.6%) • Basenji: 1/7 (14.3%) • Border Collie: 2/29 (6.9%) • Chinese Crested Dog: 2/48 (4.2%) • Danish Swedish Farmdog: 1/168 (0.6%) • German Spitz—Medium size: 1/4 (25%) • Lagotto Romagnolo: 6/637 (0.9%) • Norwegian Elkhound, grey: 46/199 (23.1%) • Old English Sheepdog: 1/55 (1.8%) • Portuguese Water Dog: 1/5 (20%) • Russian-European Laika: 1/9 (11.1%)

### Factor VII (FVII) deficiency is genetically widespread across breeds

**Fig 1:**
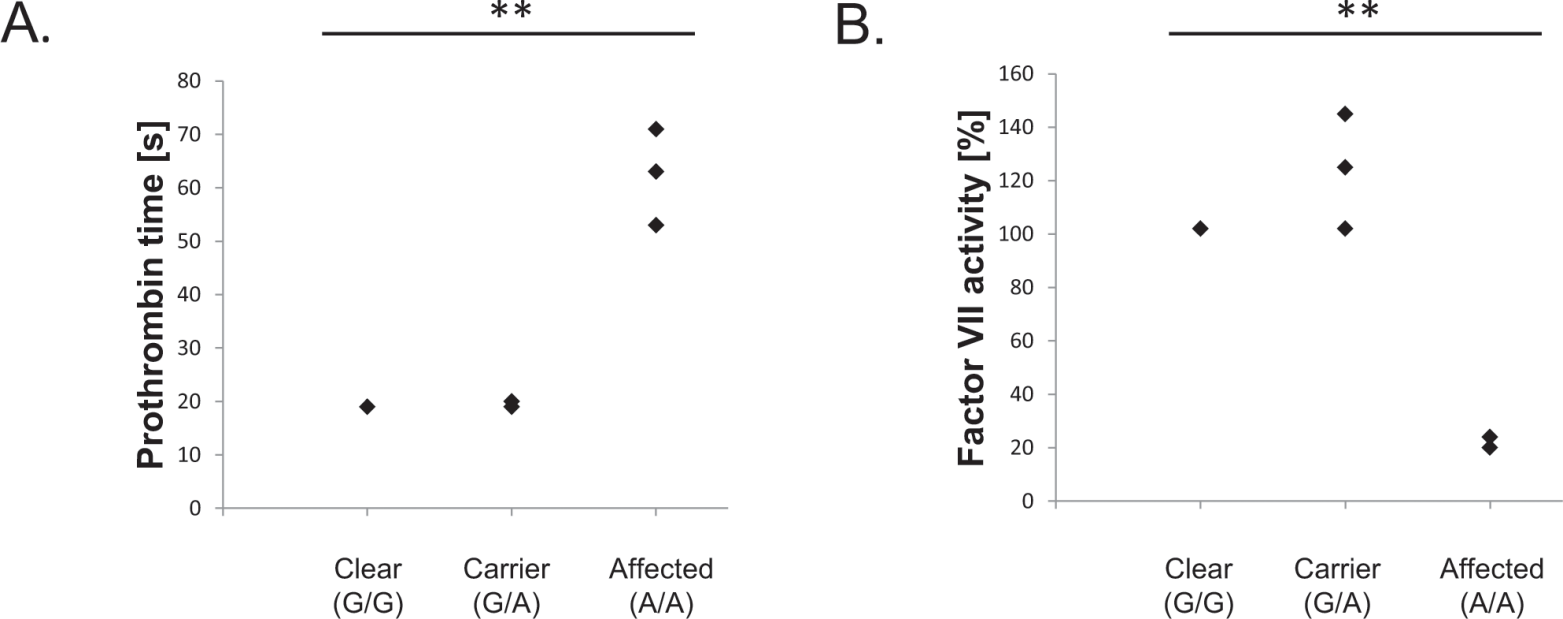
Coagulation factor VII deficiency in the Welsh Springer Spaniel. (A) Prolonged prothrombin times (t(2) = 8.21, p = 0.007) but normal partial thromboplastin times (data not shown); and (B) low plasma factor VII activity (t(3) = 9.17, p = 0.001) indicate functional factor VII deficiency and increased bleeding tendency in Welsh Springer Spaniels homozygous for the *F7* gene c.407G>A missense variant. (** P < 0.01).

Factor VII deficiency is a recessively inherited mild coagulopathy that may typically go completely unnoticed until revealed by routine coagulation screening tests or episodes of excessive bleeding due to trauma or surgery [11,12]. An *F7* missense mutation underlying canine FVII deficiency was originally characterized in research colonies of Beagles, but later also found in pet Beagles [11], Alaskan Klee Kais [12], and Scottish Deerhounds (U. Giger, unpublished). We report here ten additional breeds of varying ancestry in which the presence of the FVII deficiency-associated variant (*F7* c.407G>A) had not been previously reported (Table 1). In an effort to investigate whether the presence of the mutant allele also leads to a bleeding tendency in these additional breeds, we evaluated in vitro the coagulation in heterozygous and homozygous Welsh Springer Spaniels, and in a homozygous Finnish Hound. The three homozygous mutant Welsh Springer Spaniels dogs recruited for this study had no prior history of experiencing clinically excessive hemorrhage, but one of them exhibited marked bleeding following routine phlebotomy performed for this study. However, the mutant homozygotes had significantly prolonged prothrombin times (PT) compared to adult littermates and laboratory reference values (Fig 1A), and normal activated partial thromboplastin times (aPTT; data not shown), which is indicative of FVII deficiency. The diagnosis was further confirmed by specific FVII activity measurements on blood plasma from the same dogs (Fig 1B).

We were subsequently able to recruit one Finnish Hound homozygous for the FVII mutation for this study. Its clinical history was unremarkable, but its PT was also prolonged (72 s) compared to the reported laboratory reference values (10–20 s), indicating FVII deficiency.

### Predisposition to urate urolithiasis due to the common hyperuricosuria (HUU) mutation in Lagotto Romagnolo

**Fig 2:**
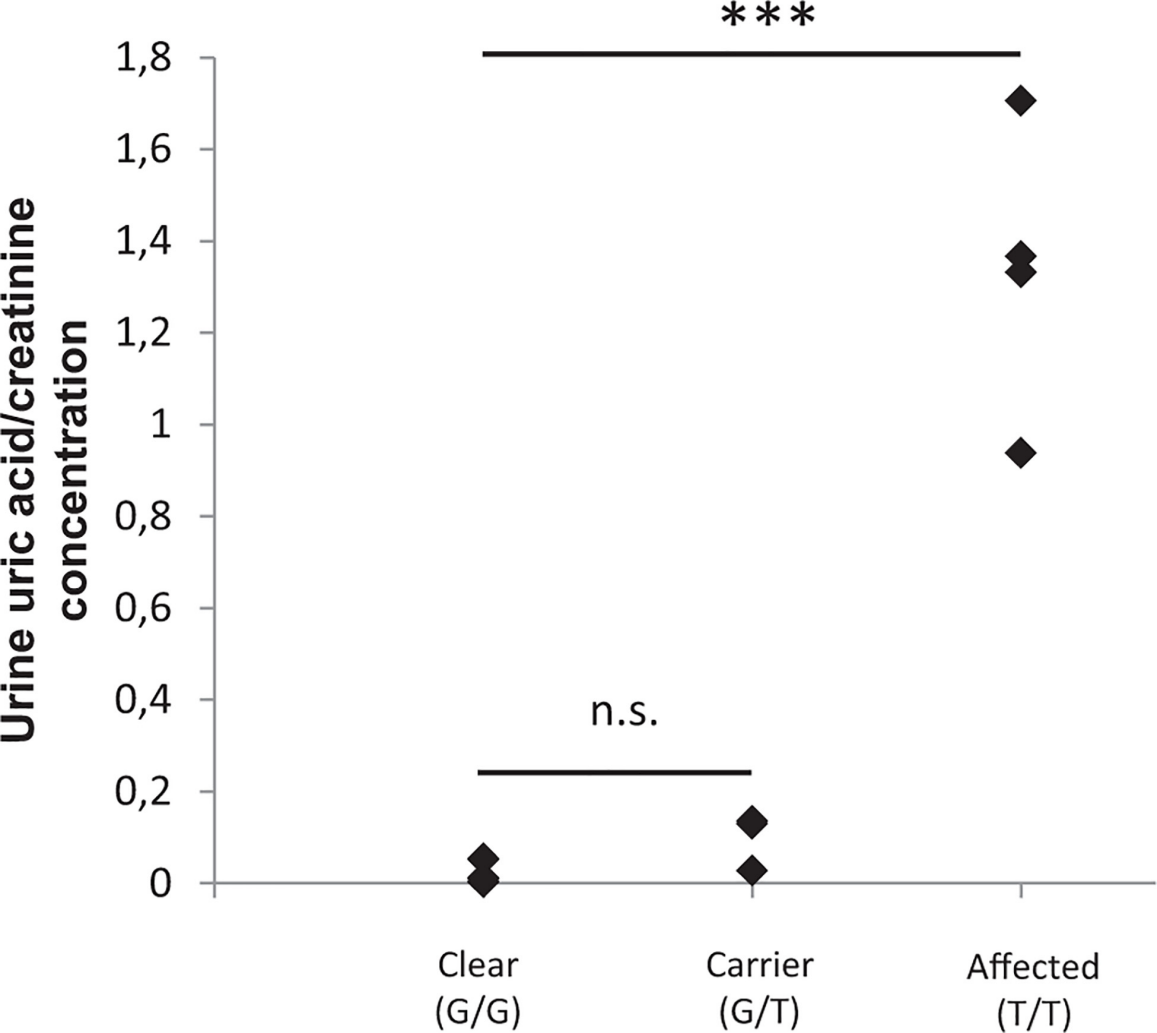
Hyperuricosuria in the Lagotto Romagnolo. Elevated urine uric acid/creatinine ratios (F(2,11) = 85.9, p = 1.93x10^-7^) in Lagotti homozygous for the *SLC2A9* gene c.616G>T variant indicate metabolic derangement and predisposition to urate stone formation. (*** P < 0.001; n.s. = not significant)

Another variant known to be common across several breeds, like the Dalmatian and Black Russian Terrier, is the *SLC2A9* gene mutation (c.616G>T) predisposing to HUU and uric acid calculi formation [10,18,21]. We confirmed the genetic presence of this risk factor in six additional breeds (Table 1) that consequently may be predisposed to complications caused by uric acid crystal formation in the urinary tract. We therefore further examined uric acid concentrations in urine samples from four Lagotto Romagnolo dogs homozygous for the risk allele, and found that all of them showed a marked increase (average 30-fold) in urine uric acid/creatinine ratio (Fig 2). One of the affected Lagotti had experienced what was described as an episode of stranguria.

### Prevalent manifestations of primary lens luxation (PLL) in Danish-Swedish Farmdogs

The *ADAMTS17* gene variant (c.1473+1G>A) associated with the development of PLL (displacement of the ocular lenses due to degeneration of zonular fibers) is prevalent in many terrier breeds [13,22]. Although some terrier ancestry is likely present in the Danish-Swedish Farmdog, no active testing of the PLL mutation had been pursued prior to the panel screening of this study. We show here that the previously reported *ADAMTS17* mutation is prevalent in the breed with a 21.9% carrier frequency (Table 1). We identified five adult dogs (2.5–4.5 years old) homozygous for the risk variant of which four were confirmed by an examining veterinarian as exhibiting clinical lens luxation in at least one eye requiring surgical treatment. The remaining homozygous dog was two and a half years old and not yet manifesting any ocular signs, but is now being closely monitored for PLL.

### Progressive early-onset cerebellar ataxia transferred between Nordic breeds

**Fig 3:**
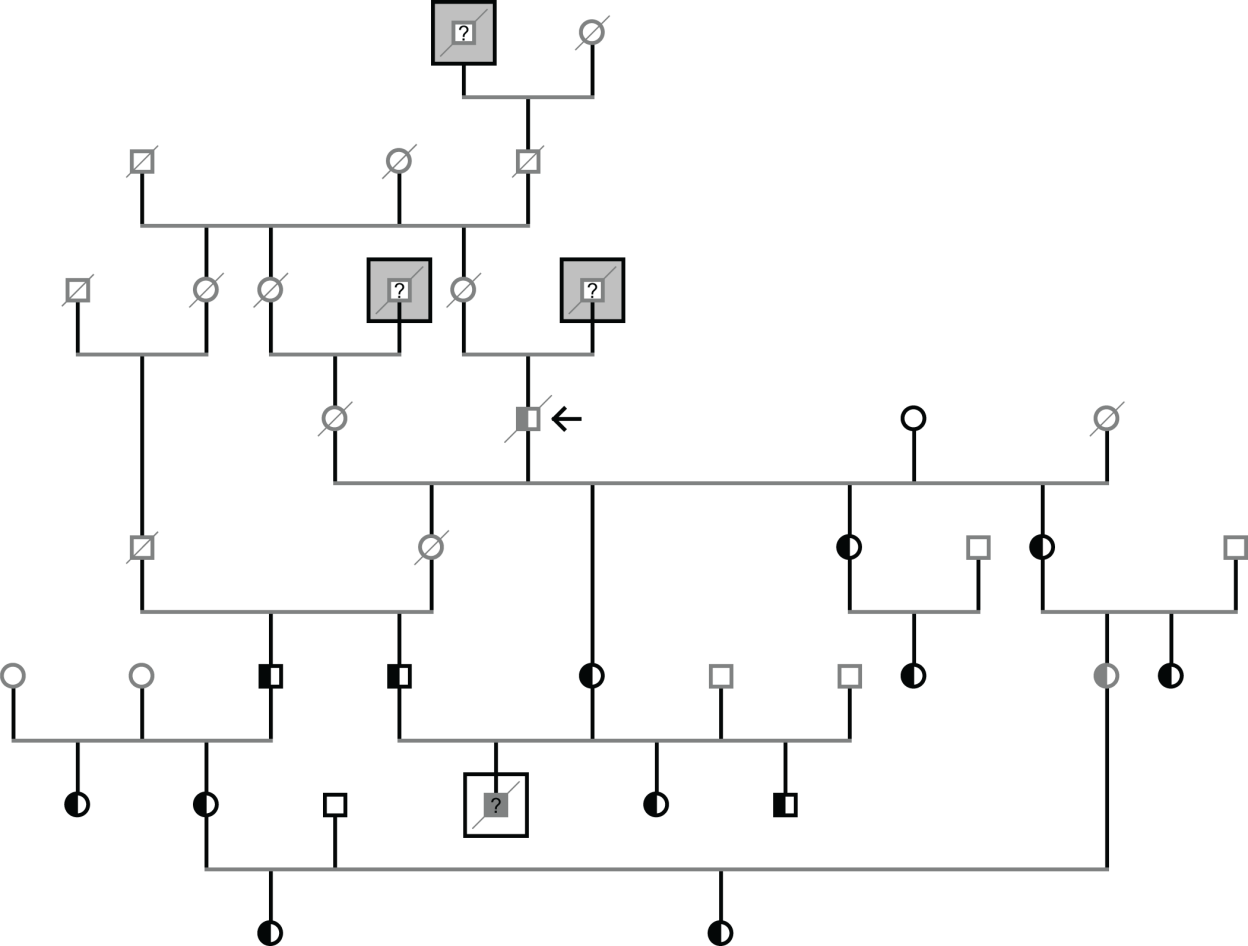
Inheritance of cerebellar ataxia in a pedigree of Norrbottenspitzes. All Norrbottenspitzes identified as carrying the Finnish Hound ataxia mutation (*SEL1L* c.1972T>C) can be traced back to the oldest obligate carrier dog (marked with an arrow symbol). Three dogs of unconfirmed background (squared on gray background) registered into the breed represent potential events of introduction of the mutation into the Norrbottenspitz populaation. A puppy (squared on white background) euthanized due to neurological symptoms matching the expected disease phenotype was born from a mating between two carrier dogs. Gender symbols of dogs with unconfirmed genotypes are shaded gray.

A rapidly progressing severe generalized cerebellar ataxia with tremors, and failure to thrive was characterized in Finnish Hound puppies [16]. Since the responsible mutation (c.1972T>C) in the *SEL1L* gene was discovered, breeders have successfully used genetic screening to eradicate the lethal disorder from the breed, and affected dogs are no longer encountered in practice. The disease variant was considered breed-specific to the Finnish Hound; yet, our panel test found carriers from another Nordic breed, the Norrbottenspitz (also known as the Nordic Spitz). The Finnish breed club describes the Norrbottenspitz population as being fairly heterogeneous with open stud books. We explored the relatedness of all thirteen confirmed carriers using the Finnish Kennel Club registry and observed that they shared ancestry to the same dogs registered into the breed in the 1990’s (Fig 3). We subsequently screened an additional separate 41 Norrbottenspitzes, all representing different unrelated litters, and observed no further carriers of the mutant allele.

Importantly, a mating of two carrier dogs was observed in the registry pedigree of the Norrbottenspitz, and one puppy from the resulting litter had been euthanized at the age of four months due to progressive neurological signs first observed at the age of nine weeks. The veterinary medical records indicated that the puppy had suffered from generalized ataxia with hypermetria, tremor, and bilateral absence of blink reflexes. A diagnosis of cerebellar cortical abiotrophy with enlarged cerebrospinal fluid compartment and sulci was confirmed by magnetic resonance imaging (MRI). No changes to the cerebrum or brainstem were detected. Although no DNA sample was available to confirm the genotype of the euthanized puppy, these clinical findings strikingly resemble those reported in Finnish Hounds with cerebellar ataxia [16].

### *ITGA10* gene mutation causes dwarfism in Chinooks

**Fig 4:**
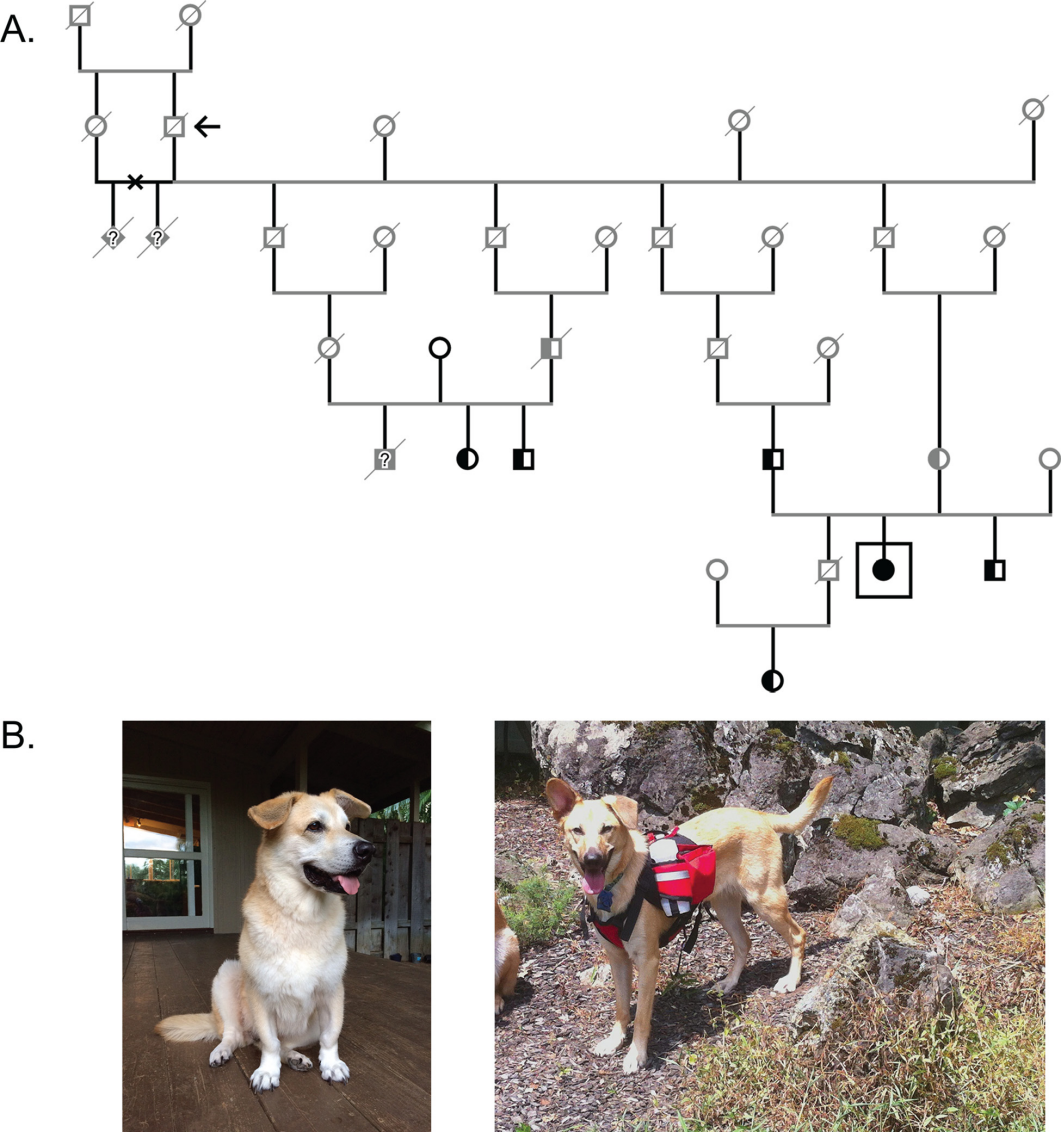
Chondrodysplasia in the Chinook. (A) Inheritance of chondrodysplasia in a pedigree of Chinooks. All carriers of the responsible variant (*ITGA10* c.2083C>T), and a genetically affected dog (squared) confirmed to have a dwarf phenotype are related to the first dwarves documented in the 1990’s as a result of a brother-sister mating (marked with an x). The mutation was likely propagated in the population through the sire of that mating (marked with an arrow), who was further bred to several other females. Gender symbols of dogs with unconfirmed genotypes are shaded gray. (B) A Chinook displaying the dwarfism phenotype (left; and squared in the pedigree) with crooked and shortened legs is shown in comparison to a normal Chinook (right).

The Chinook is a rare sled dog breed native to New England, tracing back to a male husky/mastiff-type dog with the same name born in 1917. Breeders first documented chondrodysplastic Chinooks in the 1990’s. All known cases in the breed can be traced back to a brother-sister mating producing the first known case. No conclusive information on the ancestry of these dogs exists. Intriguingly, we present an unexpected molecular explanation for one type of disproportionate dwarfism in the Chinook breed: an *ITGA10* variant (c.2083C>T) which was already known to cause disproportionate short stature dwarfism of varying severity in the Norwegian Elkhound and Karelian Bear Dog breeds [23]. This incidental finding by DNA panel screening led us to cooperate with breeders aiding in the identification of at least one chondrodysplastic Chinook dog we confirmed to be homozygous for the *ITGA10* variant, and several other carriers that are all related to the original cases from the 1990’s (Fig 4A). The homozygous mutant dog has a veterinary clinician-confirmed dwarf phenotype (Fig 4B), and early-onset hip problems and osteoarthritis were also reported.

### Familial nephropathy (FN) due to a *COL4A4* mutation in the Welsh Springer Spaniel

Presence of a hereditary renal condition resembling human Alport syndrome and known by the names familial nephropathy (FN) or autosomal recessive hereditary nephropathy (ARHN) has long been known in the English Cocker Spaniel breed [24]. Since identification of the responsible mutation (c.115A>T) in the type IV collagen gene *COL4A4*, controlled breeding and exclusions in registering offspring of known carriers have contributed to the elimination of the condition from the gene pool of the English Cocker Spaniel breed. Through DNA panel screening, we report here that the same mutation is also genetically present in several lines of the Welsh Springer Spaniel breed. Incidentally, this finding was made possible by breeders setting out to actively explore the prevalence of a far milder condition discovered by us in the breed, FVII deficiency described above. By the analysis of the pedigree registry of the Finnish Kennel Club, we confirmed that a mating between two carrier dogs had taken place, producing a puppy that had been euthanized at the age of 10 months due to chronic renal insufficiency (classified as International Renal Interest Society stage 3 of 4).

### Canine multifocal retinopathy (*cmr*)- associated variants in the Brazilian Terrier and Finnish Lapphund

Different mutations in the *BEST1* gene are known causes of canine multifocal retinopathy (*cmr*) in Mastiff-related breeds, Coton de Tulear, and Lapponian Herder [15,25]. In this study, we report the first encounter of the *cmr* type 1 (*cmr1*) variant (*BEST1* c.73C>T) in a terrier breed, the Brazilian Terrier. Moreover, a few carriers of the *cmr3* variant (*BEST1* c.1388delC) originally found in Lapponian Herder were also found in the closely related Finnish Lapphund breed.

We further explored the prevalence of *cmr1* in the Brazilian Terrier by separate genotyping in a randomly selected sample population of 233 dogs. Five mutant allele homozygotes (2.1%) and 52 carriers (22.3%) were identified, closely corresponding to the carrier frequency observed during the initial panel screening (23.4%). Of the five mutant homozygotes, three had previously undergone ophthalmology examinations or were re-examined for this study, and two were not available for clinical evaluation. We did not observe any apparent retinal changes, such as multifocal areas of retinal elevations with subretinal accumulation of serous pink-tan fluid, in the examined Brazilian Terriers homozygous for the *cmr1* variant (Table 2).

**Table 2:** Summary of ophtalmological examinations in the Brazilian Terrier.

Dog	Year of birth	Sex	Year of ophtalmology examination(s)	Findings
Dog 1	2006	Female	2008, 2014	No abnormalities
Dog 2	2007	Female	2011, 2013	No abnormalities
Dog 3	2004	Female	2008, 2014, 2015	• 2008: Distichiasis and suspected vitreous degeneration • 2014: Cortical and posterior polar cataract and vitreous degeneration • 2015: No abnormalities in the ocular fundus detected by Optical Coherence Tomography

During sequencing validation of the *cmr1* nonsense variant (*BEST1* c.73C>T; p.Arg25*), we incidentally also discovered another synonymous (silent) and therefore likely benign base substitution at the same position (c.73C>A [p.Arg25Arg]) in the several breeds (Cirneco dell’Etna, Parson Russell Terrier, Rhodesian Ridgeback, Schipperke, and St Bernard).

### Other disease variant findings in additional breeds

Beyond the findings pursued in more detail above, we found other known mutations in additional breeds as well (Table 1). Further studies will be crucial in addressing if these known variants cause a similar clinical phenotype in the additional breeds discovered. The well-known MDR1 (multi-drug resistance 1) mutation c.295_298delAGAT [26] was observed in two previously undocumented breeds, the Chinook and Danish-Swedish Farmdog. Notably, the *vWF* gene variant c.4937A>G previously associated with von Willebrand’s disease (vWD) type II [27] was present in many more breeds than currently acknowledged. A variant identified as a risk factor for craniomandibular osteopathy (CMO) in terriers [28] was also found at low frequency in other breeds. Based on the genetic findings of this study, examination of the rod-cone dysplasia 3 (rcd3; [29]) variant *PDE6A* c.1940delA as a factor explaining retinal signs in the Chinese Crested and Pomeranian breeds is warranted. The discovery of a variant (*CLCN1* c.2665insA) known to cause myotonia congenita in the Australian Cattle Dog [30] in another breed popular in Australia, the Border Collie, calls for an investigation of whether its presence is limited to a single line of dogs or is more widespread in the breed. Acatalasemia, the deficiency of catalase enzyme activity associated with a *CAT* gene variant (c.979G>A), was originally characterized in research colonies of Beagles [31,32]. In this study, we observed the variant also in a carrier American Foxhound. To our knowledge, presence and manifestation of acatalasemia due to the aforementioned *CAT* variant has not been previously documented in the pet Beagle population. We therefore note that we identified pet Beagle carriers, and a ten-month-old mutant homozygous Beagle through panel screening. During this investigation, the genetically affected Beagle developed gangrene of the oral cavity leading to the removal of three teeth at the age of eighteen months, which could be a manifestation of acatalasemia [31].

## Discussion

Through the application of a novel DNA panel screening test for nearly 100 disorders on a cohort of almost 7000 dogs, we found a high overall frequency of mutation carriers (17.8%) while discovering a sixth (15/93) of the tested disease-causing variants in additional breeds. Our findings demonstrate the power of multiplex screening as an efficient genetic testing and discovery platform for research, diagnostics and breeding. Our pioneering examples further highlight several general implications related to the approach.

First, panel screening yields new insights into the prevalence and significance of genetic risk variants across breeds. We show that several mutations are more widespread across breeds than reported in original studies with a limited sample size or breed focus, and they often–but not always–cause the same condition in the additional breeds. Therefore, there is a need for careful follow up of any unexpected discoveries. We encourage publication of such findings made during routine DNA testing, but preferably in combination with appropriate clinical evidence supporting genetic counseling and demonstrating relevance of the discovery for breeding selections and veterinary care. Within the scope of this study, we provide strong evidence supporting the clinical relevance of six known inherited conditions in additional breeds, while setting the groundwork for several future investigations.

Second, panel screening is particularly efficient when examining the breed distribution of widespread mutations likely ancient in origin. Accordingly, we present several discoveries on putative ancient variants in additional breeds (e.g., FVII, HUU, PLL, MDR1, and vWD). These disorders also represent recessively inherited conditions with clinical signs that may only be evident upon exposure to a trigger, or not primarily attributed to a specific genetic defect. One could expect such ancient variants to be readily retained in the population. We hypothesize that many more breeds harboring known ancient (and other) mutations will be discovered during future DNA screening as the numbers of tested disease variants, breeds, and dogs of each breed increase.

Third, open-minded panel screening even when no gene tests are known to be relevant for a breed *a priori* may lead to surprising discoveries that are relevant for breed health. This is exemplified by our discoveries of “Finnish Hound” cerebellar ataxia in the Norrbottenspitz, and “Norwegian Elkhound” dwarfism in the Chinook. Such findings also illustrate how inadvertent more recent transfer of mutations between breeds breaks perceived patterns of breed-specificity of genetic disease variants. Pedigree databases alone are unlikely to account fully for the molecular genetic disease heritage historically introduced into a breed. Several breed clubs are engaged in cross-breeding projects to expand the gene pool and improve the health of their breed. While these efforts are commendable, we postulate that they may further benefit from comprehensive genetic screening to ensure that known disease variants are not inadvertently introduced and subsequently enriched in the breed.

More specifically, we established presence of several disorders in additional breeds. Based on the combination of molecular, clinicopathologic and pedigree evidence presented herein, breeders and veterinarians should be aware of these conditions in their breeds and patients, respectively. FVII deficiency should be considered in Welsh Springer Spaniels and Finnish Hounds, which could exhibit bleeding tendency upon trauma or surgery. Lack of spontaneous bleeding signs in homozygous dogs highlights the mild nature of this coagulopathy, but raises concerns when combined with additional impairments of hemostasis such as hepatic disease, poisoning, and certain drug therapy. The awareness of HUU in additional breeds such as the Lagotto Romagnolo is helpful in guiding veterinarians to appropriate dietary selection, drug therapy and regular follow up for dogs at risk. Similarly, our discovery of PLL in the Danish-Swedish Farmdog has already proven crucial for proper monitoring and care of this potentially painful and severe ocular condition in dogs at risk. Swift availability of a molecular explanation for this phenotype and a validated DNA test is a relief to breeders. Based on documenting the shared ancestry of all identified carriers, we find it likely that the ataxia mutation originally found in the Finnish Hound was introduced into the Norrbottenspitz breed by registration uptake of dogs of unconfirmed heritage. Our fortunate discovery of the variant transfer has since led to active screening of the breed, and thereby prevention of the birth of further affected puppies while keeping healthy carriers in the gene pool. We further provide a molecular genetic cause for one form of dwarfism segregating in the Chinook breed, simultaneously providing an intriguing ancestral link to the Norwegian Elkhound breed.

Finally, we also established the presence of a variant known to cause FN in the English Cocker Spaniel in Welsh Springer Spaniels. Homozygosity for this variant provides a plausible retrospective explanation for renal failure in at least one puppy. Although the genotype of the puppy could not be confirmed, protein truncation by the *COL4A4* mutation, likely causing inability to combine with other collagen chains [24], makes it plausible that the pathological consequences are similar as in the original discovery breed. Truncation of the P-glycoprotein drug transporter (*ABCB1* gene) due to the well-known MDR1 mutation [26] is likely to pose a risk for severe neurotoxic side effects in any dog being administered drugs normally transported by the protein. We therefore alert veterinary clinicians to the presence of MDR1 also in the Chinook and Danish-Swedish Farmdog.

Our examination of *cmr1* eye disease serves as an example of the practical importance of two hallmarks of high fidelity panel screening: 1) confirmation of any novel finding with a second genetic technology; and 2) clinical evaluation of the relevance of the genetic finding for the breed before breeding advice is given. First, sequencing confirmation of *cmr1* findings revealed the triallelic nature of position c.73C in the *BEST1* gene. While some breeds carry the actual c.73C>T mutation, others harbor a likely harmless synonymous c.73C>A change. Awareness of this triallelic site in routine DNA testing is important, as it may lead to erroneous interpretations with some genotyping technologies, particularly if applied to mixed breed dogs. It remains to be determined whether all three alleles at this specific position are present in some breeds. Second, the clinical validation of a variant in an additional breed may end in lack of evidence for a pathological phenotype. Based on the evidence at hand, *cmr1* is unlikely to be a primary health concern in Brazilian Terriers warranting DNA testing, and carriers should not be eliminated from breeding programs. In general, the lack of a disease phenotype in additional breeds may necessitate re-evaluation of the initially reported disease-association in the original breed.

Taken together, our findings illustrate the broad potential of panel screening to: 1) advance breed health and genetics research through information on the breed distribution of mutations; 2) further pet care through preventive medicine on conditions with actionable treatment (e.g., awareness of bleeding propensity before surgery, guidance of dietary selection and monitoring in HUU, prophylactic medication and surgery in PLL, selection of safe medication based on pharmacogenetics such as *MDR1* genotype); 3) support veterinary molecular diagnostics (e.g., by provision of a plausible genetic explanation for clinical signs related to nephropathy in the Welsh Springer Spaniel); 4) prevent an emerging but still hidden severe breed health issue from spreading (e.g., ataxia in the Norrbottenspitz); and 5) effectively explain the molecular background of a breed health-related phenotype not being the primary research focus of any research group (e.g., dwarfism in the Chinook).

This is the first large scale report of DNA panel screening across purebred dogs to date, and as such we note that the investigation should be expanded to include additional disease variants and breeds in the future. Moreover, our study sample was biased towards specific breeds with active DNA testing routines and interests. We also expect some of the disease carrier frequencies reported herein to be higher than would be observed in randomly selected samples of the respective breeds due to bias generated by testing of relatives of carrier and affected dogs. The vast majority of the studied dogs represented breeds with at least one known breed-relevant test among the screened variants, and thus at least a minimum return from the testing. We believe that comprehensive DNA testing also on the remaining ~1/3 of dogs (including breeds with health concerns lacking molecular explanations, breeds lacking an active culture of DNA testing, and more rare understudied breeds) is best justified by the practical examples of novel findings in more than 30 breeds recounted in this report. The Danish-Swedish Farmdog also provides an extreme example of a breed entering the study with no known relevant DNA tests, and leaving it with four potential new conditions to consider in veterinary care and breeding. Although multiplex screening is a powerful diagnostics and research tool from the perspective of a veterinary clinician or researcher, a breeder or dog owner should not be blinded by the number of tests performed. Rather, one should think of panel screening as an accurate, convenient and cost-efficient method to obtain as many breed-relevant test results as possible with one analysis while simultaneously contributing to breed health- and canine genetics research in a novel way. Genetic counseling based on panel screening results should make it clear that the purpose of the test is not to provide a universal health certificate or give a verdict on which dog can be bred. The testing simply provides information on the specific mutations being assayed, leaving other unknown genetic and environmental health risks unexplored. Conveying these messages appropriately is particularly important when screening dogs with no prior known breed-relevant tests. Taken together, despite the need for comprehensive tools to manage the growing number of known disease mutations in breeding, the best long term solution that prevents enrichment and emergence of recessive disorders is informed breeding selections regarding known deleterious variants in combination with maintenance of genetic diversity guided by pedigree-based statistics and modern molecular genetic measurements, as previously discussed [33].

## Conclusions

We conclude that DNA multiplex screening for canine hereditary disorders represents a reliable and powerful discovery and utility tool for applications in veterinary diagnostics, genetic research, and sustainable breeding. We demonstrate that several disease-causing mutations are likely affecting and more widespread than recognized during original discovery studies due to shared breed ancestry or more recent cross-breeding. Careful genetic and clinicopathological verification to establish genotype-phenotype correlations should be part of a high quality panel screening service. Importantly, comprehensive disease screening needs to be carried out, interpreted and reported responsibly, maintaining close ties to high quality genetics research, caring veterinarians, breeders, breed health committees and kennel clubs. Panel screening, as any single DNA test, should only be viewed as one part of a breeding decision and strategy. Holistic approaches working towards overall breeding goals that are beneficial not only for the immediate health of the offspring, but also for the sustainability of the breed as a whole, are recommended.

## Supporting Information

S1 Table Number of studied dogs by breed. (XLS)

S2 Table Disease variants screened in the study. (XLS)

S3 Table Sequencing primers. (XLS)

## References

[1] Faculty of Veterinary Science, University of Sydney. Online Mendelian Inheritance in Animals, OMIA Available: http://omia.angis.org.au/.

[2] PennGen—Section of Medical Genetics, School of Veterinary Medicine, University of Pennsylvania. Listing of Canine and Feline Hereditary Disease (DNA) Testing Laboratories. Available: http://research.vet.upenn.edu/penngen/AvailableTests/TestsAvailableatLabsWorldwide/tabid/7620/Default.aspx.

[3] Slutsky J, Raj K, Yuhnke S, Bell J, Fretwell N, Hedhammar A, et al A web resource on DNA tests for canine and feline hereditary diseases. Vet J. 2013;197: 182–187. 10.1016/j.tvjl.2013.02.021 23582432 PMC3779639

[4] Miyadera K, Acland GM, Aguirre GD. Genetic and phenotypic variations of inherited retinal diseases in dogs: the power of within- and across-breed studies. Mamm Genome. 2012;23: 40–61. 10.1007/s00335-011-9361-3 22065099 PMC3942498

[5] Gultekin GI, Raj K, Foureman P, Lehman S, Manhart K, Abdulmalik O, et al Erythrocytic pyruvate kinase mutations causing hemolytic anemia, osteosclerosis, and seconday hemochromatosis in dogs. J Vet Intern Med. 2012;26: 935–944. 22805166 10.1111/j.1939-1676.2012.00958.x PMC3650904

[6] Brons AK, Henthorn PS, Raj K, Fitzgerald CA, Liu J, Sewell AC, et al SLC3A1 and SLC7A9 mutations in autosomal recessive or dominant canine cystinuria: a new classification system. J Vet Intern Med. 2013;27: 1400–1408. 10.1111/jvim.12176 24001348 PMC3946761

[7] Forman OP, De Risio L, Mellersh CS. Missense mutation in CAPN1 is associated with spinocerebellar ataxia in the Parson Russell Terrier dog breed. PLoS One. 2013;8: e64627 10.1371/journal.pone.0064627 23741357 PMC3669408

[8] Gilliam D, O'Brien DP, Coates JR, Johnson GS, Johnson GC, Mhlanga-Mutangadura T, et al A homozygous KCNJ10 mutation in Jack Russell Terriers and related breeds with spinocerebellar ataxia with myokymia, seizures, or both. J Vet Intern Med. 2014;28: 871–877. 10.1111/jvim.12355 24708069 PMC4238845

[9] Zeng R, Coates JR, Johnson GC, Hansen L, Awano T, Kolicheski A, et al Breed distribution of SOD1 alleles previously associated with canine degenerative myelopathy. J Vet Intern Med. 2014;28: 515–521. 10.1111/jvim.12317 24524809 PMC4238831

[10] Karmi N, Brown EA, Hughes SS, McLaughlin B, Mellersh CS, Biourge V, et al Estimated frequency of the canine hyperuricosuria mutation in different dog breeds. J Vet Intern Med. 2010;24: 1337–1342. 10.1111/j.1939-1676.2010.0631.x 21054540 PMC5535792

[11] Callan MB, Aljamali MN, Margaritis P, Griot-Wenk ME, Pollak ES, Werner P, et al A novel missense mutation responsible for factor VII deficiency in research Beagle colonies. J Thromb Haemost. 2006;4: 2616–2622. 16961583 10.1111/j.1538-7836.2006.02203.x

[12] Kaae JA, Callan MB, Brooks MB. Hereditary factor VII deficiency in the Alaskan Klee Kai dog. J Vet Intern Med. 2007;21: 976–981. 17939552 10.1892/0891-6640(2007)21[976:hfvdit]2.0.co;2

[13] Gould D, Pettitt L, McLaughlin B, Holmes N, Forman O, Thomas A, et al ADAMTS17 mutation associated with primary lens luxation is widespread among breeds. Vet Ophthalmol. 2011;14: 378–384. 10.1111/j.1463-5224.2011.00892.x 22050825

[14] Parker HG, Kukekova AV, Akey DT, Goldstein O, Kirkness EF, Baysac KC, et al Breed relationships facilitate fine-mapping studies: a 7.8-kb deletion cosegregates with Collie eye anomaly across multiple dog breeds. Genome Res. 2007;17: 1562–1571. 17916641 10.1101/gr.6772807 PMC2045139

[15] Guziewicz KE, Zangerl B, Lindauer SJ, Mullins RF, Sandmeyer LS, Grahn BH, et al Bestrophin gene mutations cause canine multifocal retinopathy: a novel animal model for best disease. Invest Ophthalmol Vis Sci. 2007;48: 1959–1967. 17460247 10.1167/iovs.06-1374 PMC1931491

[16] Kyostila K, Cizinauskas S, Seppala EH, Suhonen E, Jeserevics J, Sukura A, et al A SEL1L mutation links a canine progressive early-onset cerebellar ataxia to the endoplasmic reticulum-associated protein degradation (ERAD) machinery. PLoS Genet. 2012;8: e1002759 10.1371/journal.pgen.1002759 22719266 PMC3375262

[17] Genoscoper Laboratories Oy. MyDogDNA Technical Sheet—Design, Technology, and Performance Data. Available: http://mydogdna.com/sites/default/files/files/mydogdna_technical_sheet.pdf.

[18] Karmi N, Safra N, Young A, Bannasch DL. Validation of a urine test and characterization of the putative genetic mutation for hyperuricosuria in Bulldogs and Black Russian Terriers. Am J Vet Res. 2010;71: 909–914. 10.2460/ajvr.71.8.909 20673090 PMC5551899

[19] GenoPro. Available: http://www.genopro.com.

[20] Suomen Kennelliitto ry. KoiraNet jalostustietojärjestelmä. Available: http://jalostus.kennelliitto.fi/.

[21] Bannasch D, Safra N, Young A, Karmi N, Schaible RS, Ling GV. Mutations in the SLC2A9 gene cause hyperuricosuria and hyperuricemia in the dog. PLoS Genet. 2008;4: e1000246 10.1371/journal.pgen.1000246 18989453 PMC2573870

[22] Farias FH, Johnson GS, Taylor JF, Giuliano E, Katz ML, Sanders DN, et al An ADAMTS17 splice donor site mutation in dogs with primary lens luxation. Invest Ophthalmol Vis Sci. 2010;51: 4716–4721. 10.1167/iovs.09-5142 20375329

[23] Kyostila K, Lappalainen AK, Lohi H. Canine chondrodysplasia caused by a truncating mutation in collagen-binding integrin alpha subunit 10. PLoS One. 2013;8: e75621 10.1371/journal.pone.0075621 24086591 PMC3783422

[24] Davidson AG, Bell RJ, Lees GE, Kashtan CE, Davidson GS, Murphy KE. Genetic cause of autosomal recessive hereditary nephropathy in the English Cocker Spaniel. J Vet Intern Med. 2007;21: 394–401. 17552442 10.1892/0891-6640(2007)21[394:gcoarh]2.0.co;2

[25] Zangerl B, Wickstrom K, Slavik J, Lindauer SJ, Ahonen S, Schelling C, et al Assessment of canine BEST1 variations identifies new mutations and establishes an independent bestrophinopathy model (cmr3). Mol Vis. 2010;16: 2791–2804. 21197113 PMC3008713

[26] Mealey KL, Bentjen SA, Gay JM, Cantor GH. Ivermectin sensitivity in collies is associated with a deletion mutation of the mdr1 gene. Pharmacogenetics. 2001;11: 727–733. 11692082 10.1097/00008571-200111000-00012

[27] Kramer JW, Venta PJ, Klein SR, Cao Y, Schall WD, Yuzbasiyan-Gurkan V. A von Willebrand's factor genomic nucleotide variant and polymerase chain reaction diagnostic test associated with inheritable type-2 von Willebrand's disease in a line of german shorthaired pointer dogs. Vet Pathol. 2004;41: 221–228. 15133170 10.1354/vp.41-3-221

[28] Hytonen MK, Arumilli M, Lappalainen AK, Owczarek-Lipska M, Jagannathan V, Hundi S, et al Molecular Characterization of Three Canine Models of Human Rare Bone Diseases: Caffey, van den Ende-Gupta, and Raine Syndromes. PLoS Genet. 2016;12: e1006037 10.1371/journal.pgen.1006037 27187611 PMC4871343

[29] Petersen-Jones SM, Entz DD, Sargan DR. cGMP phosphodiesterase-alpha mutation causes progressive retinal atrophy in the Cardigan Welsh corgi dog. Invest Ophthalmol Vis Sci. 1999;40: 1637–1644. 10393029

[30] Finnigan DF, Hanna WJ, Poma R, Bendall AJ. A novel mutation of the CLCN1 gene associated with myotonia hereditaria in an Australian cattle dog. J Vet Intern Med. 2007;21: 458–463. 17552451 10.1892/0891-6640(2007)21[458:anmotc]2.0.co;2

[31] Fukuda K, Shindo H, Yamasita K, Mizuhira V. Catalase activity of erythrocytes from beagle dog: an appearance of hereditary acatalasemia. Acta Histochem Cytochem. 1982;15: 685–690.

[32] Nakamura K, Watanabe M, Takanaka K, Sasaki Y, Ikeda T. cDNA cloning of mutant catalase in acatalasemic beagle dog: single nucleotide substitution leading to thermal-instability and enhanced proteolysis of mutant enzyme. Int J Biochem Cell Biol. 2000;32: 1183–1193. 11137458 10.1016/s1357-2725(00)00057-1

[33] Farrell LL, Schoenebeck JJ, Wiener P, Clements DN, Summers KM. The challenges of pedigree dog health: approaches to combating inherited disease. Canine Genet Epidemiol. 2015;2: 3-015-0014-9. eCollection 2015. 10.1186/s40575-015-0014-9 PMC4579364 26401331

